# Supplementary material for: Epidermal barrier defects link atopic dermatitis with altered skin cancer susceptibility
Source: eLife. 2014 May 6;3:e01888. doi: 10.7554/eLife.01888 (PMC4007207; doi:10.7554/eLife.01888)
Supplement: Supplementary file 1. — Primers and TaqMan probes used. DOI: http://dx.doi.org/10.7554/eLife.01888.012 [file elife01888s001.docx]

Primers and Taqman probes used:

| **Gene** | **Forward Primer** | **Reverse Primer** | **TaqMan Probe** |
| --- | --- | --- | --- |
| AhR | AGGACCAAACACAAGCTAGA | TGGAGATCTCGTACAACACA |  |
| AhRR | GCCAATGCTGTCTAATGAAG | AACAGAGCACCAAGAAAACA |  |
| Bax | CTCACGGAGGAAGTCCAGTGT | CATGTTTGCTGATGGCAACTT |  |
| Bcl-xl | CTGCTCACTTACTGGGTCTGC | AAGAGGCGGATGAAACAATTC |  |
| Cxcl1 | ACCCGCTCGCTTCTCTGT | GCTCTGGATGTTCTTGAGGTG |  |
| CxcL13 |  |  | [Mm04214185_s1](https://products.appliedbiosystems.com:443/ab/en/US/adirect/ab?cmd=ABAssayDetailDisplay&assayID=Mm04214185_s1&Fs=y&adv_phrase3=EXACT&adv_phrase2=EXACT&adv_phrase1=EXACT&assayType=GE&catID=601267&adv_kw_filter3=ALL&srchType=keyword&adv_kw_filter2=ALL&SearchRequest.Common.QueryText=cxcl13&kwdropdown=ge&adv_kw_filter1=ALL&species=Mus+musculus&adv_query_text3=&searchType=keyword&adv_query_text2=&adv_query_text1=&uploadType=ID+List&adv_boolean3=AND&adv_boolean2=AND&adv_boolean1=AND&chkBatchQueryText=false&kwfilter=ALL&SearchRequest.Common.PageNumber=1&msgType=ABGEKeywordResults) |
| Cxcl2 | CAGACTCCAGCCACACTTCA | CACACTCAAGCTCTGGATGTTC |  |
| Cxcl5 | TGAAAAACCAGAAGGAGGTCTG | GCATTCCGCTTAGCTTTCTTT |  |
| CYP1A1 |  |  | Mm00487218_m1 |
| CYP1B1 |  |  | Mm00487229_m1 |
| GAPDH | aacatcaaatggggtgaggcc | gttgtcatggatgaccttggc | Mm99999915_g1 |
| GAPDH |  |  | Mm00487218_m1 |
| g-GAPDH | TGGTTCACACCCATCACAAACA | GGTGAAGGTCGGTGTGAACGG |  |
| GMCSF |  |  | [Mm01290062_m1](https://products.appliedbiosystems.com:443/ab/en/US/adirect/ab?cmd=ABAssayDetailDisplay&assayID=Mm01290062_m1&Fs=y&adv_phrase3=EXACT&adv_phrase2=EXACT&adv_phrase1=EXACT&assayType=GE&catID=601267&adv_kw_filter3=ALL&srchType=keyword&adv_kw_filter2=ALL&SearchRequest.Common.QueryText=GM-CSF&kwdropdown=ge&adv_kw_filter1=ALL&species=Mus+musculus&adv_query_text3=&searchType=keyword&adv_query_text2=&adv_query_text1=&uploadType=ID+List&adv_boolean3=AND&adv_boolean2=AND&adv_boolean1=AND&chkBatchQueryText=false&kwfilt) |
| H60 | AGATTTCAGTTGCTGCCTCA | ACATGTGCAGCAGTGGTTG |  |
| Hes-1 | GGAAATGACTGTGAAGCACCTC | GATCTGGGTCATGCAGTTGG |  |
| Hes-5 | GTGGAGATGCTCAGTCCCAAG | AGCTTCAGCTGCTCTATGCTG |  |
| Hey-1 | TAGTGAGCTGGACGAGACCAT | GTCTTTTCCTGGCCAAAACCT |  |
| Hey-2 | AGTAGCTGCTCCTCCTTCGTC | GGTAGTTGTCGGTGAATTGGA |  |
| HRas-mut61 | CTAAGCCTGTTGTTTTGCAGGAC | CATGGCACTATACTCTTCTA |  |
| IL12p40 |  |  | Mm00434174_m1 |
| IL13 |  |  | Mm00434204_m1 |
| IL17α | ACCGCAATGAAGACCCTGAT | CATCTTCTCGACCCTGAAAGTGA |  |
| IL18 | ACTTTGGCCGACTTCACTGTA | CTTCACAGAGAGGGTCACAGC |  |
| IL1α |  |  | Mm00439620_m1 |
| IL1β | AGCTCATATGGGTCCGACAG | GACCTTCCAGGATGAGGACA |  |
| IL22 | CATGCAGGAGGTGGTACCTT | CAGACGCAAGCATTTCTCAG | Mm00444241_m1 |
| IL33 | TCCTGCCTCCCTGAGTACATA | GACTTGCAGGACAGGGAGACT |  |
| IL4 | TGGACTCATTCATGGTGCAG | AACATGGGAAAACTCCATGC |  |
| IL6 |  |  | Mm00446190_m1 |
| IFNγ | TCTGGAGGAACTGGCAAAAG | GCTGATGGCCTGATTGTCTT |  |
| Jag-1 | TGGCACCACTATGTCCTGGAG | ACTGGTATCGGTGCGAATGTG |  |
| Mdm2 | TCTGTATCGCTTTCTCCTGTCTG | ACGATGGCGTAAGTGAGCATT |  |
| Notch-1 | ACCTGTGACCTGCTCACTCTC | GGCAGCGACAGATGTATGAAG |  |
| Notch-2 | TGCCAATACTCCACCTCTCAC | CATTTTCGCAGGGATGAGATA |  |
| Notch-3 | CTCGTATTGGCAGTGTGTGC | CCTGTCCACCTGGTTATGGA |  |
| P53 exon4 | TAGGCTGAGAACACAGTCCTGAGG | GCATTGAAAGGTCACACGAAAGAC |  |
| P53 exon5 | CCTGATCGTTACTCGGCTTGTC | CAACTGTCTCTAAGACGCACAAACC |  |
| P53 exon8/9 | GCAGATATGACAAGAGGGGTTG | GCGAGAGACAGAGGCAATAAGG |  |
| p53 | AACCGCCGACCTATCCTTAC | CTTCTGTACGGCGGTCTCTC |  |
| Rae-1 | CTAGTGCCACCTGGGAATTCA | CATCATTAGCTGATCTCCAGCTCA |  |
| RAGE |  |  | Mm00545815m1 |
| S100A8 |  |  | [Mm00496696_g1](https://products.appliedbiosystems.com:443/ab/en/US/adirect/ab?cmd=ABAssayDetailDisplay&assayID=Mm00496696_g1&Fs=y&adv_phrase3=EXACT&adv_phrase2=EXACT&adv_phrase1=EXACT&assayType=GE&catID=601267&adv_kw_filter3=ALL&srchType=keyword&adv_kw_filter2=ALL&SearchRequest.Common.QueryText=S100a8&kwdropdown=ge&adv_kw_filter1=ALL&species=Mus+musculus&adv_query_text3=&searchType=keyword&adv_query_text2=&adv_query_text1=&uploadType=ID+List&adv_boolean3=AND&adv_boolean2=AND&adv_boolean1=AND&chkBatchQueryText=false&kwfilt) |
| S100A9 |  |  | [Mm00656925_m1](https://products.appliedbiosystems.com:443/ab/en/US/adirect/ab?cmd=ABAssayDetailDisplay&assayID=Mm00656925_m1&Fs=y&adv_phrase3=EXACT&adv_phrase2=EXACT&adv_phrase1=EXACT&assayType=GE&catID=601267&adv_kw_filter3=ALL&srchType=keyword&adv_kw_filter2=ALL&SearchRequest.Common.QueryText=S100a9&kwdropdown=ge&adv_kw_filter1=ALL&species=Mus+musculus&adv_query_text3=&searchType=keyword&adv_query_text2=&adv_query_text1=&uploadType=ID+List&adv_boolean3=AND&adv_boolean2=AND&adv_boolean1=AND&chkBatchQueryText=false&kwfilt) |
| Survivin | TCTGGCAGCTGTACCTCAAG | ATCAGGCTCGTTCTCGGTAG |  |
| TNFα | CAGGCTTGTCACTCGAATTTT | CAAATGGCCTCCCTCTCAT |  |
| TSLP | TCTCAGGAGCCTCTTCATCCT | CTCACAGTCCTCGATTTGCTC |  |
| VEGFα | ATGGGACTTCTGCTCTCCTTC | CATGAACTTTCTGCTCTCTTGG |  |
